# In Vivo Healthy Benefits of Galacto-Oligosaccharides from *Lupinus albus* (LA-GOS) in Butyrate Production through Intestinal Microbiota

**DOI:** 10.3390/biom11111658

**Published:** 2021-11-09

**Authors:** Lucila A. Godínez-Méndez, Carmen M. Gurrola-Díaz, José Sergio Zepeda-Nuño, Natali Vega-Magaña, Rocio Ivette Lopez-Roa, Liliana Íñiguez-Gutiérrez, Pedro M. García-López, Mary Fafutis-Morris, Vidal Delgado-Rizo

**Affiliations:** 1 Departamento de Fisiología, CIINDE, Centro Universitario de Ciencias de la Salud, Universidad de Guadalajara, Guadalajara 44340, Jalisco, Mexico; ariadnegodinez@gmail.com (L.A.G.-M.); lilianainiguez11@gmail.com (L.Í.-G.); mfafutis@gmail.com (M.F.-M.); 2 Departamento de Biología Molecular y Genómica, Centro Universitario de Ciencias de la Salud, Universidad de Guadalajara, Guadalajara 44340, Jalisco, Mexico; carmenhpv@yahoo.de; 3 Departamento de Microbiología y Patología, Centro Universitario de Ciencias de la Salud, Universidad de Guadalajara, Guadalajara 44340, Jalisco, Mexico; jsergio.zepeda@academicos.udg.mx (J.S.Z.-N.); alejandra.vega@academicos.udg.mx (N.V.-M.); 4 Departamento de Farmacobiología, Centro Universitaro de Ciencias Exactas e Ingenierias, Universidad de Guadalajara, Guadalajara 44430, Jalisco, Mexico; rocio.lopez@academicos.udg.mx; 5 Departamento de Botánica y Zoología, Centro Universitario de Ciencias Biologíco y Agropecuarias, Universidad de Guadalajara, Guadalajara 45200, Jalisco, Mexico; macedonio54@gmail.com

**Keywords:** *Lupinus albus*, short chain fatty acids, ulcerative colitis, intestinal microbiota, dysbiosis, goblet cells, butyryl-CoA transferase gene

## Abstract

Animal digestive systems host microorganism ecosystems, including integrated bacteria, viruses, fungi, and others, that produce a variety of compounds from different substrates with healthy properties. Among these substrates, α-galacto-oligosaccharides (GOS) are considered prebiotics that promote the grow of gut microbiota with a metabolic output of Short Chain Fatty Acids (SCFAs). In this regard, we evaluated *Lupinus albus* GOS (LA-GOS) as a natural prebiotic using different animal models. Therefore, the aim of this work was to evaluate the effect of LA-GOS on the gut microbiota, SCFA production, and intestinal health in healthy and induced dysbiosis conditions (an ulcerative colitis (UC) model). Twenty C57BL/6 mice were randomly allocated in four groups (*n* = 5/group): untreated and treated non-induced animals, and two groups induced with 2% dextran sulfate sodium to UC with and without LA-GOS administration (2.5 g/kg bw). We found that the UC treated group showed a higher goblet cell number, lower disease activity index, and reduced histopathological damage in comparison to the UC untreated group. In addition, the abundance of positive bacteria to butyryl-CoA transferase in gut microbiota was significantly increased by LA-GOS treatment, in healthy conditions. We measured the SCFA production with significant differences in the butyrate concentration between treated and untreated healthy groups. Finally, the pH level in cecum feces was reduced after LA-GOS treatment. Overall, we point out the in vivo health benefits of LA-GOS administration on the preservation of the intestinal ecosystem and the promotion of SCFA production.

## 1. Introduction

The gut microbiome is a complex natural ecosystem that is composed of fungi, viruses, parasites, archaea, and bacteria [1]. Bacteria are around 90% of the total gut microbiota [2,3]. Five phyla primarily compose the taxonomy of the intestinal bacteria. Firmicutes and Bacteroidetes are the major phyla including more than 90% of all microbiota [4]. Proteobacteria, Actinobacteria, and Verrucomicrobia phyla constitute the remaining percentage [5]. In the host, the intestinal health depends on the gut microbiota balance, Short Chain Fatty Acid (SCFA) production, tight junction protein regulation, and pH levels, among others [6,7,8].

Intestinal homeostasis is achieved through diverse functions of gut microbiota like nutrients and energy supply to the intestine, inhibition of pathogens colonization, balance pro-inflammatory and anti-inflammatory mediators, and production of metabolites that promote a healthy environment [9,10]. On the other hand, intestinal health may be disrupted by a loss of proportion in the microbiota phyla [11]. This effect is known as dysbiosis and is generally associated with inflammation and damage to the intestine [12]. Dysbiosis is characterized by a loss of diversity, structure, or function of the gut microbiota [13].

Several diseases are related to a dysbiotic state, including inflammatory bowel disease (IBD), obesity, diabetes, autoimmunity, allergy, and cancer [14]. In particular, a modification in the phyla distribution is clearly associated with the pathogenesis of Ulcerative Colitis (UC), a disease that is included in the IBD [15]. Indeed, the functional diversity and stability of intestinal bacteria are impaired in the intestinal microbial population from UC patients [16]. Previously, it was reported that UC is characterized by a reduction of Firmicutes and an increase of Bacteroidetes and facultative anaerobes [17]. In addition to the gut microbiota, other factors have been implicated in the development of UC, including a dysregulated immune response, genetic susceptibility, and environmental factors [15].

The UC is characterized by mucosal inflammation that initiates in the rectum and extends proximally in the colon [18]. Various experimental mice models have been development to investigate the pathogenesis of UC [19]. The most commonly induced experimental colitis is by dextran sulfate sodium (DSS). This model is simple and affords a high degree of uniformity and reproducibility of most lesions in the distal colon [20]. 

The diagnosis of UC in humans and in experimental models considers clinical symptoms. The pharmacological management of UC includes amino-salicylates (5-ASA), corticosteroids, and thiopurines [21]. However, corticosteroids can produce severe secondary effects, including osteoporosis, depression, type 2 diabetes mellitus, and cataracts [22]. Therefore, therapeutic alternatives are necessary to complement the pharmacological treatment and reverse the dysbiosis process and foment a healthy ecosystem in the intestine.

In recent years, prebiotics have been used as nutritional complements to modulate the gut microbiota ecosystem [23]. Prebiotics are classically defined as “a substrate that is selectively utilized by host microorganism conferring a health benefit” [24]. Taking into consideration the concept of a prebiotic described by the Association for Probiotics and Prebiotics (ISAPP), new compounds, such as nanoparticles, can participate as prebiotic sources [25]. The most evaluated prebiotics are fructo-oligosaccharides (FOS), galacto-oligosaccharides (GOS), and cyclodextrins (CDs) [26]. 

Prebiotics produce several beneficial effects exerted by the gut microbiota on the host. Particularly, it has been observed that populations from *Bifidobacterium*, *Lactobacillus*, and lactic acid bacteria increase after prebiotics administration [27,28]. Furthermore, these bacteria utilized as principal source hexose and pentoses come from prebiotics and transform it into pyruvate through the Embden–Meyerhof–Parnassian or pentose-phosphate pathways. 

The end products of fermentation include the SCFAs and formic and lactic acids [29]. The SCFA production is related to intestinal health. In fact, SCFAs participate in many important functions in the intestine. Acetate, propionate, and butyrate are the three major SCFAs produced during bacterial fermentation. These acids promote the energy supply for enterocytes, the regulation of the expression and distribution of tight junction proteins as well as the maintenance of the goblet cell population and its subsequent mucus production, and the restoration of the integrity of the intestinal barrier [30].

Patients with IBD show reduced levels of total SCFAs in feces [31]. In the UC experimental model, the butyric acid was significantly decreased [32]. Lower butyrate concentration is associated with the permeability of epithelium, loss of the tight junction proteins, and inflammation mediated via NF-κB, among others [33,34]. New approaches encourage the use of prebiotics to promote butyrate production to mitigate the damage of UC [34].

Prebiotics are contained in plants and are now used to improve human nutrition. Some edible plants rich in oligosaccharides include lupin, chicory, asparagus, and garlic, among others [26]. Lupin is an important legume that has been consumed for centuries by inhabitants of the Mediterranean and Andean regions [35]. Lupin’s crop protein is important both as food for animals and in human nutrition and as a nutraceutical source [36]. There exist many scientific reports about lupin proteins indicating antihypertensive, hypoglycemic, and anti-hypercholesteremic effects [37,38]. In addition, lupins, like other legumes, contain soluble carbohydrates—for instance sucrose, raffinose, stachyose, and verbascose [39]. 

These oligosaccharides belonging to the GOS family and are formed by 1–6 galactosides linked to C6 of the glucose moiety of sucrose [39]. GOS from *Lupinus albus* (LA-GOS) possess prebiotic proprieties that promote the growth of gut bifidobacterial and Lactobacillus present in the large intestine of mammals with the production of SCFAs [40]. These compounds propitiate an environment that allows the grow of benefit microbiota that avoid infection by pathogenic bacteria [41]. The effects of LA-GOS in the gut microbiota have only been evaluated in an in vitro ecosystem with *Lupinus albus* seeds. In this study, *L. albus* increased the SCFA production, in the same manner as FOS and analogous to LA-GOS [42].

Prebiotic Sources (GOS, FOS, xylitol, and arabinoxylans) have been shown to have a positive effect in the modulation of intestinal microbiota, such as in Lupinus species [43,44,45]. Nevertheless, GOS from *Lupinus albus* have not been evaluated in the maintenance of gut microbiota balance and its intestinal effects. Considering the relevant role of prebiotics in preserving natural ecosystems, like the gut microbiota and its related effects on SCFA production, we aimed to evaluate the effect of GOS from *L. albus* under healthy and dysbiotic conditions, including experimental UC.

## 2. Materials and Methods

### 2.1. Extraction of GOS from Lupinus albus

*Lupinus albus* flour was obtained from dehulled seeds, ground to 2 mm particle size, and defatted with hexane. The α-galactooligosaccharides extraction was carried out as described previously by Gulewicz and cols. [46]. Briefly, 100 g of defatted flour was extracted with 200 mL of 50% ethanol (*v*/*v*) at 40 °C overnight. After extraction, the supernatant was decanted. The flour was reextracted with fresh 50% ethanol under the same conditions, and the supernatants from two cycles of extraction were combined. The clear supernatant was concentrated on a rotary vacuum evaporator (BÜCHI, Essen, Germany) at 50 °C to the volume of 25 mL, placed in glass separator, and dropped into 100% ethanol with continuous stirring. 

The ratio of water extract volume to volume of 100% ethanol should be 1:10. The crude raffinose family oligosaccharides (RFOs) precipitate was separated from supernatant by centrifugation at 3000 rpm for 15 min. The RFOs precipitate was then placed into a vacuum desiccator overnight in order to remove any ethanol residue. The proximate composition, protein, moisture, ether extract, ash, and crude fiber contents of the *L. albus* defatted flour were determined as described in the official methods of analysis from AOAC International.

### 2.2. Animals

Twenty eight-week-old male C57BL/6 mice weighing between 18 and 25 g were obtained from the University Center for Health Sciences (CUCS) Bioterium of the University of Guadalajara. The experimental animals were maintained in a standard laboratory, with the following standard conditions: 25 °C temperature, 55.0 ± 5% humidity, and a 12-h light–dark cycle with ad libitum access to water and a standard rodent diet (Purina LabDiet^®^ 5001). The ethics committee from the University Center of Health Sciences (CUCS), University of Guadalajara, approved this protocol with a registration number CI-00420 (approved on 14 February 2020). All animal procedures were conducted according to the production, care, and use of laboratory animals established in the Mexican Official Standard (NOM-062-ZOO-1999).

### 2.3. Preparation of Dosage Alpha-Galactooligosaccharides and Treatment

The dosage selection of *Lupinus albus* α-galactooligosaccharides (LA-GOS), used in the treatment, was determined according to the literature [47,48,49,50,51,52]. To select the therapeutic dosage, we considered the following theoretical aspects: the evaluated experimental model, its demonstrated effect in the model, and the dosage conversion according to the selected species (mice model). Finally, we decided to use a 2.5 g/kg-bw per day dosage Figure S1. Therefore, LA-GOS treatment consisted of the daily oral administration of 2.5 g/kg-bw/day of LA-GOS dissolved in 200 μL of sterile water during fourteen consecutive days (from day 1 to 14).

### 2.4. Experimental Design

Experimental ulcerative colitis induction in mice was performed by giving drinking water *ad libitum* containing 2% (*w*/*v*) dextran sulfate sodium (DSS) M_r_ ~40,000 (# 42867-Sigma Aldrich, St. Louis, MO, USA) during seven consecutive days (from day 7 to 14) [53]. After one acclimation week, 20 mice were randomly distributed in four experimental groups (*n* = 5): the control group (Healthy), the healthy treated group (Healthy + LA-GOS, 2.5 g/kg bw/day)), the model group (untreated Ulcerative colitis-UC model), and the treated UC model group (UC + LA-GOS, 2.5 g/kg bw/day). 

Mice of the healthy and healthy + LA-GOS groups were given distilled water without DSS during the whole experimental period. The UC and UC + LA-GOS groups were induced into ulcerative colitis on the 7th day, for one week (from day 7 to 14). In the healthy + LA-GOS and UC + LA-GOS groups, the LA-GOS treatment was administered by oral gavage during 14th consecutive days, whereas the healthy and UC groups received only the vehicle of treatment (sterile water) by gavage for fourteen consecutive days. 

After the experimental period, all mice were sacrificed on the 15th day [48]. The mice were anesthetized, and blood was collected from the internal iliac vein. Euthanasia of the laboratory animals was performed by cervical dislocation. From each experimental animal, the colon was extracted from the cecum to rectum. In addition, the mesenteric tissue was removed from the colonic tissue. Then, the colon tissue was rinsed with normal saline solution (0.9% *w*/*v*), and the colon length was measured and documented. Descending colon tissue was fixed immediately in 10% formaldehyde in 1X phosphate buffer saline (PBS) to perform later histological analysis.

### 2.5. Disease Activity Index (DAI)

To evaluate the ulcerative colitis severity, the Disease Activity Index (DAI) was determined at UC basal day (day 7) and at the end of the experiment (day 14). The DAI was calculated based on the clinical signs scoring including weight loss, stool character, and the presence of blood in the feces Table 1 [54].

### 2.6. Histopathological Analysis

The descending colon tissue was excised, washed in saline solution, fixed in 10% formaldehyde in 1X phosphate buffer saline (PBS), embedded in paraffin, and sectioned (5 μm thick). Later, the colonic tissue was stained for seven minutes with Mayer’s Hematoxylin (HYCEL, Zapopan, Mexico) and then with 0.2% eosin (HYCEL, Zapopan, Mexico) for one minute. The severity of the epithelial damage was evaluated by a certified pathologist with the following criteria Table 2 [55]:

### 2.7. Goblet Cells Count

Paraffin blocks of the descending colon tissue were cut into 5 μm sections. For each sample, one slide containing five tissue sections was prepared. The sections were stained with Alcian blue (HYCEL, Zapopan, Mexico) and counterstained with hematoxylin to visualize the goblet cells. The number of goblet cells per each sample was obtained from three randomly selected sections of colonic epithelium. From each selected section, ten crypts were randomly chosen (30 crypts in total, per sample). The goblet cells quantity was measured using the viewing APERIO Image Scope software (https://www.leicabiosystems.com/es/patologia-digital/gestion/aperio-imagescope/) by LEICA with a 20× magnification (100 μm). Therefore, the number of goblet cells was expressed as “number of goblet cells per crypt per 100 µm” [56,57].

### 2.8. Feces Protocol

The fecal samples were collected on the 14th day in all experimental groups. To guarantee a homogeneous quality of the sample, all fresh fecal samples were collected in sterile tubes, and immediately after, frozen in dry ice and stored at −80 °C until analysis. In the same manner, before the sacrifice of the animals (15th day), the feces from cecum were collected in sterile tubes and immediately frozen in dry ice and stored at −80 °C until analysis [58].

### 2.9. Relative Abundance of Microbial Phyla and Butyryl CoA Transferase Using Semiquantitative Real-Time PCR

To quantify the microbial phyla and butyryl-CoA transferase gene positive bacteria, genomic DNA was extracted from 25 mg of fecal samples of the 14th day using a Quick-DNA Fecal/Soil kit (Zymo Research, Irvine, CA, USA) according to the manufacturer’s instructions. DNA concentrations were measured by nanodrop (Thermo Fisher Scientific, Waltham, MA, USA). The quantification via real-time PCR was performed using Maxima SYBR Green Master Mix (Thermo Fisher Scientific, #K0251, Waltham, MA, USA). All experiments were realized with the StepOne Thermocycler (Applied, Biosystem, Waltham, MA, USA). 

Primers 27F: 5′-AGAGTTTGATCMTGGCTCAG-3′ and 519R: 5′-GWATTACCGCGGCKGCTC-3′ were used to amplify the V3 to V4 regions of the 16S rDNA of total bacteria [59]. The reaction was performed under the following conditions: hold 95 °C (7 min) followed by 25 cycles of 95 °C (30 s), 60 °C (50 s), and 72 °C (40 s). To determine the microbiota phyla, the used primers were: Firmicutes Firm934F: 5′-GGAGYATGTGGTTTAATTCGAAGCA-3′ and Firm1060R: 5′-AGCTGACGACAACCATGCAC3′. Bacteroidetes Bact934F: 5′-GGAGYATGTGGTTTAATTCGAAGCA-3′ Bact1060R: 5′ AGCTGACGACAACCATGCAG-3′ [60]. Proteobacteria F: 5′ TCGTCAGCTCGTGTYGTGA-3′ and R: 5′ CGTAAGGGCCATGATG-3′ [61]. 

Reactions were realized under the following conditions: hold 95 °C (10 min), followed by 35 cycles of 95 °C (30 s), alienation temperature Firmicutes 69 °C, Bacteroidetes 67 °C, and Proteobacteria 63 °C (1 min) and 72 °C (40 s) of extension for all phyla. In addition, Butyryl-CoA transferase gene positive bacteria were amplified using the following primers BCoATsxrF: 5′-GCIGAICATTTCACITGGAAYWSITGGCAYATG and BCoATscrR: 5′-CCTGCCTTTGCAATRTCIACRAANGC-3′ [62]. The analysis was done under the following experimental conditions: hold 95 °C (5 min), followed by 40 cycles of 95 °C (30 s), 53 °C (30 s), and 72 °C (30 s). 

All primer sequences were analyzed by the BLAST program of the National Center for Biotechnology Information, to verify the correct alignment with the target. Relative quantification of PCR products was determined with the 2^ΔΔCt^ method [63]. The analysis of 16s rDNA for total bacteria was used as a reference gene. For each analyzed gene, a melting curve analysis was performed to confirm amplicons.

### 2.10. Short Chain Fatty Acids Quantification

For the SCFAs quantification, 25 mg of feces from the cecum of each sample were weighed in a sterile tube, and 200 μL of sterile distilled water was added and homogenized. The next step was added to the homogenate 200 μL of the solution prepared with N-butanol, tetrahydrofuran, and acetonitrile in a 50:30:20 ratio. After that, it was aggregated with 40 μL HCl 0.1M, 20 mg citric acid, and 40 mg of NaCl. All the components were mixed until their homogenization. The supernatant was obtained by centrifugation at 13,000× *g* for 10 min. 

The supernatant was filtered with a Whatman GD/X syringe filter 0.22 μm of PVDF (MERCK, Billerica, MA, USA). Afterwards, the supernatant was analyzed in a Shimadzu GC 2010 plus gas chromatograph with a flame ionization detector (FID) (Shimadzu Scientific Instrument, Kyoto, Japan). To perform the analysis, a high polarity stationary phase column (Mega-Acid) was used, with the following characteristics: 30 m × 0.25 mm of intern diameter (id) and thickness: 0.40 μm. 

The conditions of analysis were injection 3 μL, split 1:25, oven temperature 250 °C, detector temperature 250 °C, gas flow: N_2_ 30 mL/min, H_2_ 40 mL/min, and air 400 mL/min. The data were analyzed with the LabSolutions, Chromatography Data System software (Shimadzu Scientific Instruments, Kyoto, Japan). In addition, the quantifications of each acid (acetic acid, butyric acid, and propionic acid) were analyzed with a standard curve of six points (100, 50, 25, 12.5, 6.25, and 3.125 ppm). The concentration of SCFAs in each sample was determined by the interpolation of the data with the standard curve [64].

### 2.11. pH Measurement

In each sample, the pH value was measured in feces collected from the cecum. For the preparation of the sample, 50 mg of feces were weighed in a 500 μL sterile tube, and 200 μL of sterile water was added and vortexed for 2 min [65]. The pH level was determined using a pre-calibrated potentiometer with a ROSS BNWP 8220 microelectrode (Thermo Fisher Scientific, Waltham, MA, USA). This electrode is specifically designed for the pH measurement of small volumes and in dirty samples, such as feces.

### 2.12. Immunohistochemistry

The Claudin-1 protein expression was determined in descending colon tissue. The resected tissue was fixed immediately with 10% formaldehyde in 1X phosphate buffer saline (PBS), processed and embedded in paraffin. Paraffin-embedded samples were cut, and sections (3 µm) were treated with the Novolink polymer detection system according to the manufacturer’s instructions. Antigen retrieval was performed with EDTA buffer (1 mM EDTA, 0.05% Tween 20, and pH 8.0) for 40 min. 

Sections were incubated overnight at 4 °C with a mouse monoclonal primary antibody (sc-166338, Santa Cruz Biotechnology, Danvers, MA, USA) at a 1:250 dilution. Adipose tissue was used as a negative control. Finally, the chromogen working solution and hematoxylin were used to perform the detection and counterstain, respectively. The claudin-1 expression was documented using the Aperio LV1 IVD equipment and Aperio imagescope software (Leica Biosystem, Chicago, IL, USA). The staining intensity was scored as follows: 0 = negative, 1 = weak, 2 = moderate, and 3 = strong [66].

### 2.13. Statical Analysis

Data are presented as the mean ± standard error of the mean (SEM) for the relative abundance of microbiota phyla and butyryl-CoA transferase gene. The DAI score, Length colon, histopathological score, number of goblet cells, staining intensity score, SCFAs, and pH levels data are expressed as the mean ± standard deviation (SD). The Brown–Forsythe test was performed to evaluate the change in the relative abundance of Bacteroidetes, Proteobacteria-phyla and butyryl-CoA transferase genes. The Kruskal–Wallis test was utilized to assess the expression of claudin-1. In addition, one way ANOVA was employed to analyze the DAI, length colon, histopathological score, number of goblet cells, total bacteria, Firmicutes phylum, SCFAs concentrations, and pH levels. All statistical analyses were performed with the GraphPad Prism software (version 8.0, San Diego, CA, USA). A *p* value ≤ 0.05 was considered significant.

## 3. Results

### 3.1. LA-GOS Treatment Ameliorates the Clinical Characteristics of Ulcerative Colitis

**Figure 1:**
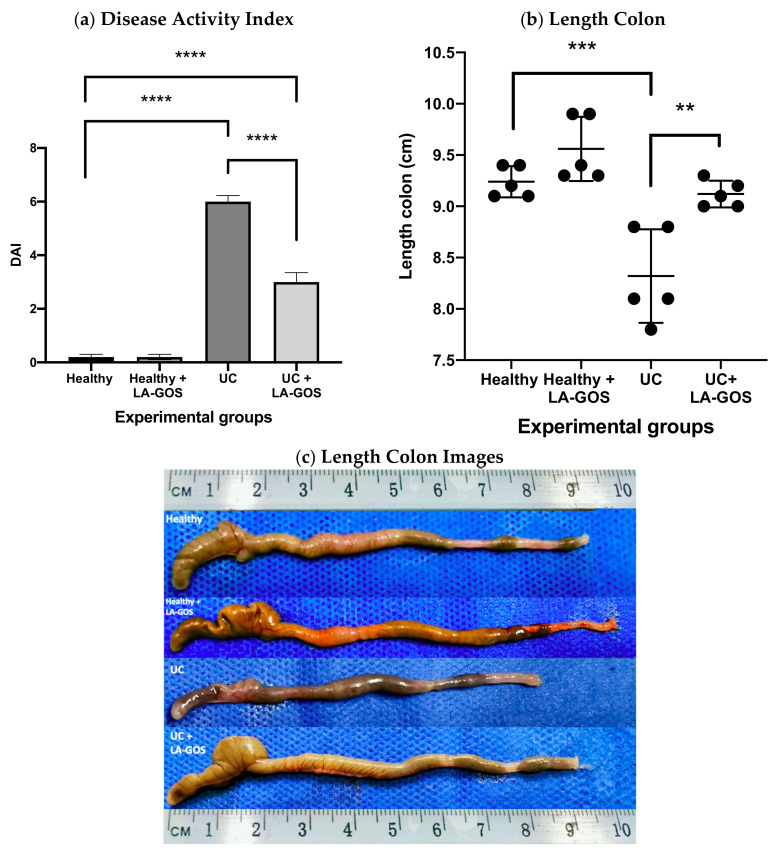
(**a**) The disease activity index (DAI) was determined as the average of the score of weight loss, stool consistency, and bleeding at the 14th day (previously described). The mean value of DAI was significantly different when the UC and UC + LA-GOS groups were compared to the healthy group. Indeed, UC + LA-GOS significantly reduced the DAI in comparison to the UC group. (**b**) Length of the colon (cecum until rectum) is represented in cm. Differences were found between the healthy and UC groups. In fact, the LA-GOS treatment increased the length colon of UC induced animals when they were compared against the untreated UC group. (**c**) Representative images of the colons from each experimental group are shown. Data are expressed as the mean ± SD. A one-way ANOVA test was used to analyze the DAI and length colon data, followed by Dunnett’s comparison test. (** *p* ≤ 0.01, *** *p* ≤ 0.001, and **** *p* ≤ 0.0001).

As shown in Figure 1a, the Disease Activity Index (DAI) was reduced by 50% after LA-GOS treatment in the UC model in comparison with the untreated UC group. The colon length was similar in the healthy and healthy + LA-GOS experimental groups (9.2 ± 0.15 cm and 9.6 ± 0.3 cm, respectively) (Figure 1b,c). As expected, the colon length in the UC group was significantly reduced in comparison with the healthy group (8.3 ± 0.45 cm vs. 9.2 ± 0.15 cm, *p* < 0.001). On the contrary, the colon length in the UC + LA-GOS group remained a comparable length as the healthy group (9.12 ± 0.13 vs. 9.2 ± 0.15 cm) (Figure 1b,c). We can suggest that the LA-GOS treatment prevented the shortening of the colon, which was evident when comparing the treated and untreated UC groups (9.12 ± 0.13 vs. 8.3 ± 0.45 cm, *p* ≤ 0.01).

### 3.2. LA-GOS Administration Reduced the Histopathological Colon Damage and Increased the Goblet Cell Number

**Figure 2:**
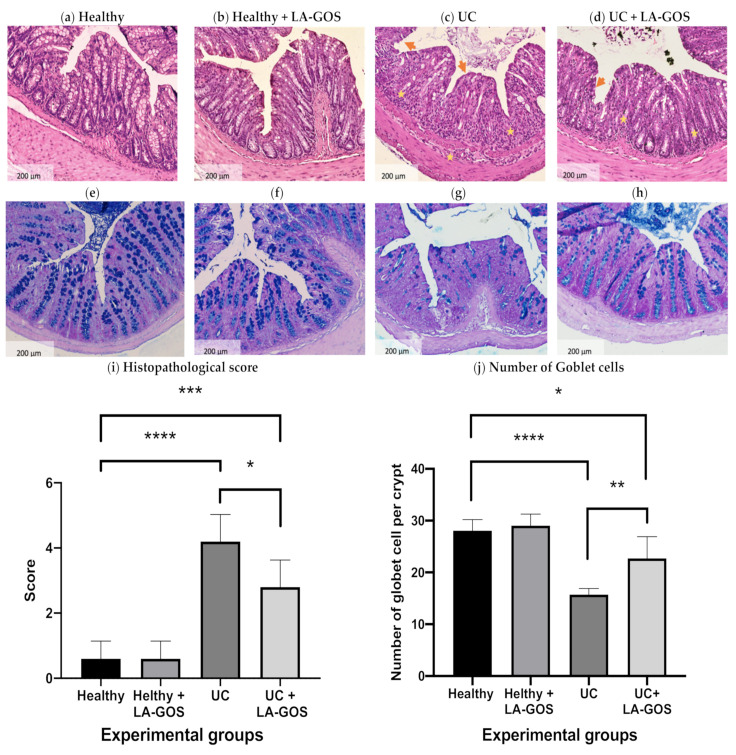
Histological images stained with H&E (**a**–**d**) and Blue Alcian (**e**–**h**) representing each experimental group are shown (×10 magnification, bar= 200 μm). (**a**,**e**) Healthy group, (**b**,**f**) Healthy + LA-GOS, (**c**,**g**) Ulcerative Colitis (UC), and (**d**,**h**) UC + LA-GOS. (**a**,**b**) Healthy and healthy + LA-GOS showed normal histological characteristics. (**c**) UC group showed severe inflammatory cells in the mucosa and submucosa (yellow stars) and erosion (orange arrows) in the descendent colon. LA-GOS administration attenuated DSS-induced microscopic colon damage. (**d**) Inflammatory cells in mucosa (yellow stars) and erosion (orange arrows). The evaluation of the goblet cell number was normal (**e**,**f**), on the contrary, a loss of goblet cells was observed in the UC group (**g**). After LA-GOS treatment, the number of goblet cells was improved (**h**). A semiquantitative analysis was performed to evaluate the histopathological score (**i**) and the number of goblet cells per crypt (**j**). Semiquantitative analysis revealed differences in the mean value of the histopathological score among UC, UC + LA-GOS and healthy groups. Moreover, UC + LA-GOS significantly reduced the histopathological score in comparison to the UC group. Differences in the number of goblet cells per crypt were found between healthy and UC groups. In addition, the LA-GOS treatment augmented the goblet cell number in comparison with those found in the untreated UC group. Data are exhibited as the mean ± SD. A one-way ANOVA test was used to evaluate the histopathological score and the number of goblet cells, followed by Dunnett’s multiple comparison test. (* *p* ≤ 0.01, ** *p* ≤ 0.005, *** *p* ≤ 0.0005, and **** *p* ≤ 0.0001).

Histopathological changes in colitis were analyzed and scored in the descendent colon tissue. Representative H&E-stained histological sections as well as the histopathological score are shown in Figure 2a–d,i. The colon from both, healthy and healthy + LA-GOS groups, showed healthy epithelium, that means the presence of well-organized crypts, non-infiltration in mucosa and submucosa, and no ulceration or tissue erosion. 

In contrast, the colon epithelium from the UC group developed severe inflammatory lesions, characterized by complete loss or crypts, surface erosion, and extensive diffuse ulceration as well as mucosa and submucosa infiltrates (*p* ≤ 0.0001). Notably, the UC + LA-GOS group exhibited a significant reduction in inflammation and colon injury. After treatment, we observed mild inflammation, preservation of the majority of crypts, surface erosion, and only focal ulceration and infiltrates uniquely in mucosa (*p* ≤ 0.0005). Based on the histopathological score, LA-GOS treatment improves the intestine health in comparison with the untreated UC group (*p* ≤ 0.01).

We use the Alcian staining in order to visualize the goblet cells, and after that these cells exhibit a blue color. Alcian-positive cells were observed inside of the crypts from descending colonic tissue (Figure 2e–g). A semiquantitative analysis of the goblet cell number per crypt was performed and is shown in Figure 2j. As described in the literature [67], the goblet cell number was significantly reduced after the DSS-induced colitis model (44% in comparison with the healthy group, *p* ≤ 0.0001). On the other hand, LA-GOS treatment increased the goblet cell number by 25% in the UC model compared to the untreated UC group (*p* ≤ 0.005). These results show that LA-GOS treatment prevented the reduction of goblet cells, and this indicates that the mucosal damage could be attenuated in ulcerative colitis.

### 3.3. Claudin-1, a Tight Junction Protein, Is Reduced in DSS-Induced Acute Colitis

**Figure 3:**
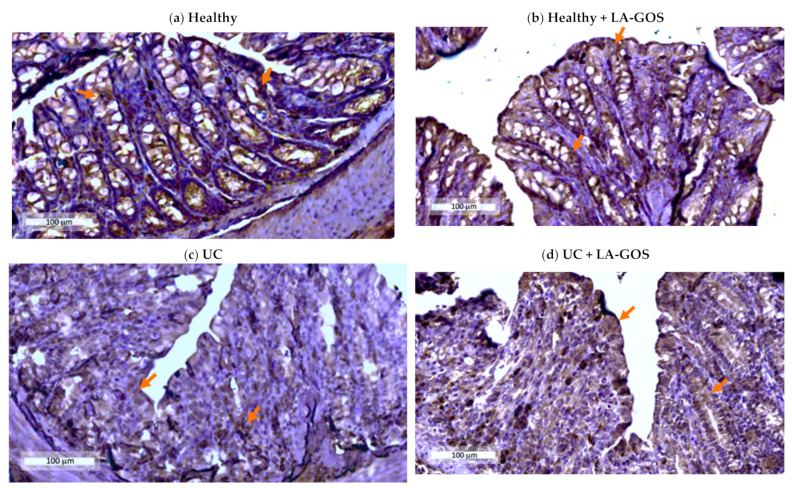

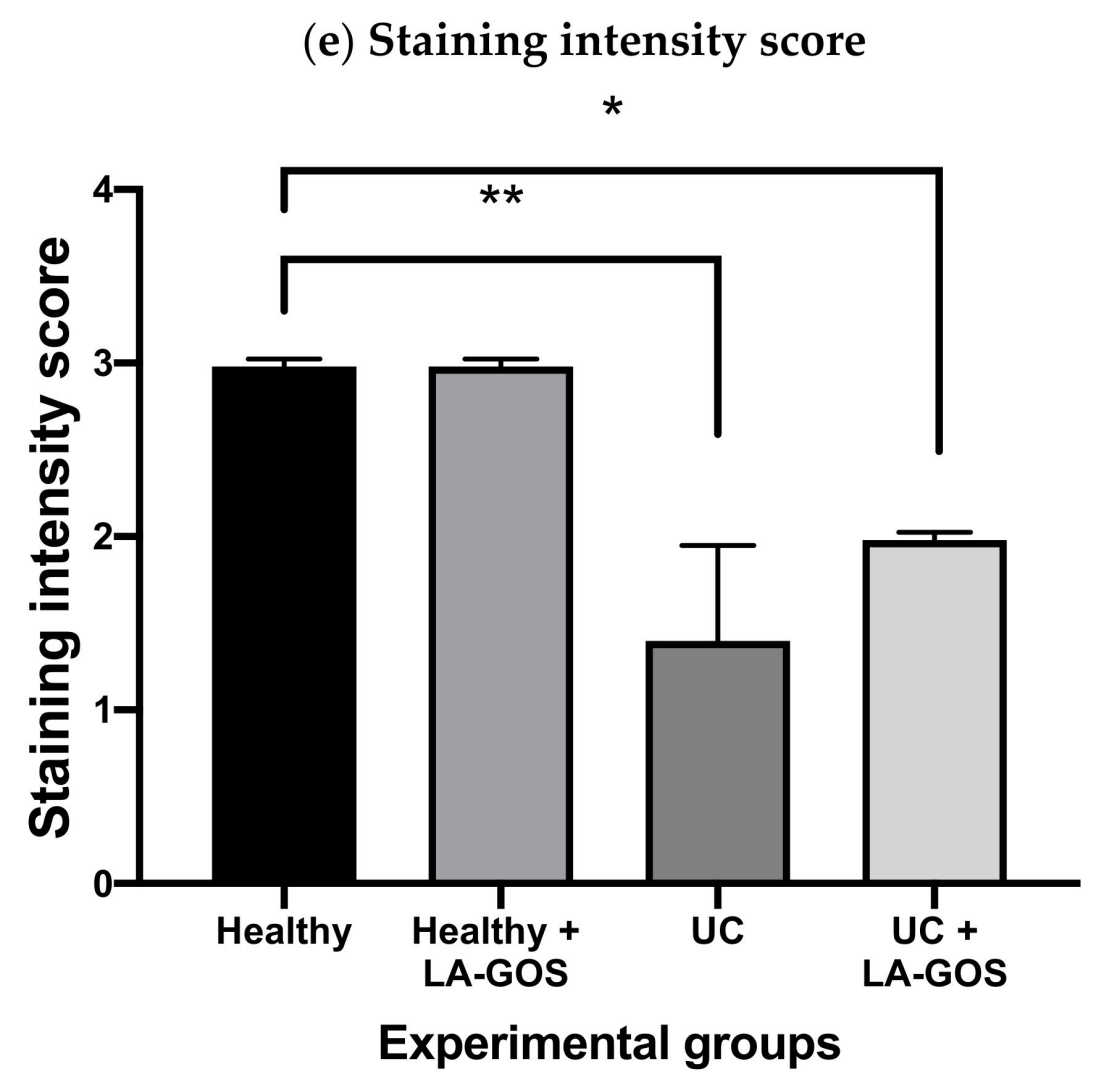
Immunohistochemistry (IHC) was performed for the expression of the claudin-1 protein in the descendent colon tissue of each experimental group. (**a**–**d**) Representative images for claudin-1 counterstained with Hematoxylin are shown (×20. magnification, bar= 100 μm). Claudin-1 is localized at the crypt surface and basolateral membrane (orange arrows) (**a**) Healthy, (**b**) Healthy + LA-GOS, (**c**) Ulcerative colitis (UC), (**d**) UC + LA-GOS. In the healthy and healthy + LA-GOS groups was found a normal expression of claudin-1 (**a**,**b**), whereas, in the UC and UC + LA-GOS groups, the expression decreased (**c**,**d**). (**e**) Semiquantitative score of claudin 1 protein levels. Data are expressed as the mean ± SD. Semiquantitative analysis revealed differences in the mean value of the immunohistochemistry score among UC and UC + LA-GOS vs. healthy group. The Kruskal–Wallis test was used to compare the claudin-1 IHC score followed by Dunn’s test. (* *p* ≤ 0.05 and ** *p* ≤ 0.005).

In order to understand the effect of administration of LA-GOS treatment on the expression of tight junctions, claudin-1 Inmunohistochemistry was performed in descending colon tissue (Figure 3a–e). The Claudin-1 expression was significantly decreased in both UC groups in comparison with the healthy group (*p* ≤ 0.05 and *p* ≤ 0.005). LA-GOS treatment shows a tendency to recover the claudin-1 expression in the treated UC group (20% more than the untreated group, without significance).

### 3.4. Gut Microbiome Shift in Mice with DSS-Induced Acute Colitis and Treated with LA-GOS

**Figure 4:**
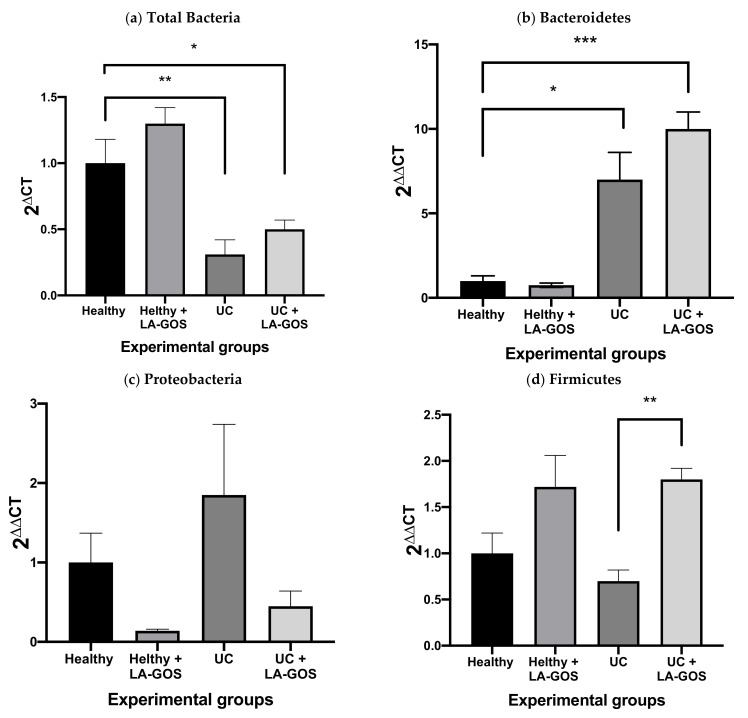
Intestinal microbiota were analyzed to determine the proportion of total bacteria and the proportion of the phyla-Bacteroidetes, Proteobacteria, and Firmicutes in feces at 14th day. Relative quantification (real-time PCR) of the total bacteria was performed by 16s rRNA analysis (2^Δ^^CT^). Each phylum proportion was calculated by 2^ΔΔ^^CT^ analysis between total bacteria and specific phylum. (**a**) Relative abundances of the total bacteria. Statical differences were found between heathy and UC groups. (**b**) Relative abundance of the Bacteroidetes phylum. The mean values of Bacteroidetes were significantly different when UC and UC + LA-GOS groups were compared against to the healthy group. (**c**) Relative abundance of the Proteobacteria phylum. The healthy + LA-GOS and UC + LA-GOS groups showed a tendency to decrease the proportion of Proteobacteria phylum; however, these differences were not statically significant. (**d**) Relative abundance of the Firmicutes phylum. LA-GOS treatment significantly increased the proportion of Firmicutes in the UC treated group. Results are expressed as the mean ± SEM. One way ANOVA was performed to analyze the total bacteria and Firmicutes phylum, followed by the Dunnett’s multiple comparison test (* *p* = 0.05 and ** *p* = 0.005). The Brown–Forsythe test was performed to analyze the phyla Bacteroidetes and Proteobacteria, followed by Dunnett’s T3 multiple comparison test. (* *p* ≤ 0.05, ** *p* = 0.01, and *** *p* = 0.001).

To further explore the protective effect of LA-GOS treatment, we quantified the gut microbiota by real-time PCR. The relative abundance of total bacteria was determined by the 16s rDNA gene. In our study, we found a significant reduction of the total bacteria in both UC groups in comparison with the healthy group (* *p* ≤ 0.05 and ** *p* ≤ 0.005, respectively). However, LA-GOS treatment exerts a 19% gain of the relative abundance of total bacteria when compared with the untreated UC group Figure 4a. Moreover, we determined the relative abundance of the Bacteroidetes phylum with significant differences between the healthy group and both UC groups (* *p* ≤ 0.05, *** *p* = 0.001) Figure 4b. 

Interestingly, we observed that LA-GOS treatment increased, by 30%, the relative abundance of the Bacteroidetes phylum in the UC + LA-GOS group when it was compared against the untreated UC group. With respect to the Proteobacteria phylum, LA-GOS treatment reduced the relative abundance of the Proteobacteria phylum in UC conditions Figure 4c (data not significant). Finally, when we evaluated the Firmicutes phylum, LA-GOS treatment increased the relative abundance by 110% in comparison with the UC untreated group (** *p* = 0.01). All these results show the promissory effect of LA-GOS as a gut microbiota modulator.

### 3.5. LA-GOS Increased the Proportion of Butyryl-CoA Transferase Gene Positive Bacteria in the Healthy Group

**Figure 5:**
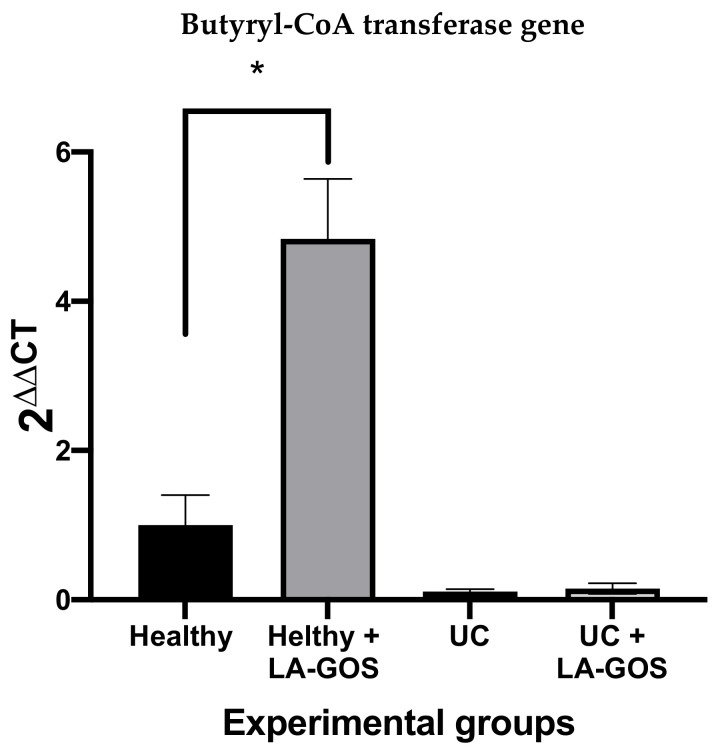
The Butyryl-CoA transferase (*But*) gene was quantified in the total bacteria in feces at the 14th day. The *But* gene was relative quantified using 2^ΔΔ^^CT^ analysis. Data analysis revealed significant differences between the healthy and healthy + LA-GOS groups. The results are expressed as the mean ± SEM. The Brown–Forsythe test was performed to analyze the data, followed by Dunnett’s T3 multiple comparison test (* *p* ≤ 0.01).

Butyryl-CoA transferase is the most important pathway utilized by the gut microbiota to the production of Butyric acid [68]. In our study, we evaluated the effect of LA-GOS treatment on the relative abundance of bacteria, which contain the butyryl CoA transferase (*But*) gene. In a healthy condition, we found that LA-GOS treatment significantly increases the abundance of *But* gene positive bacteria (*p* ≤ 0.01). By contrast, under the UC environment, the abundance of positive bacteria to the *But* gene is reduced. Nevertheless, after LA-GOS treatment in these conditions, it was not possible to restore the initial *But* positive bacteria number (Figure 5).

### 3.6. LA-GOS Modified the Production and Pattern of Short Chain Fatty Acids

**Figure 6:**
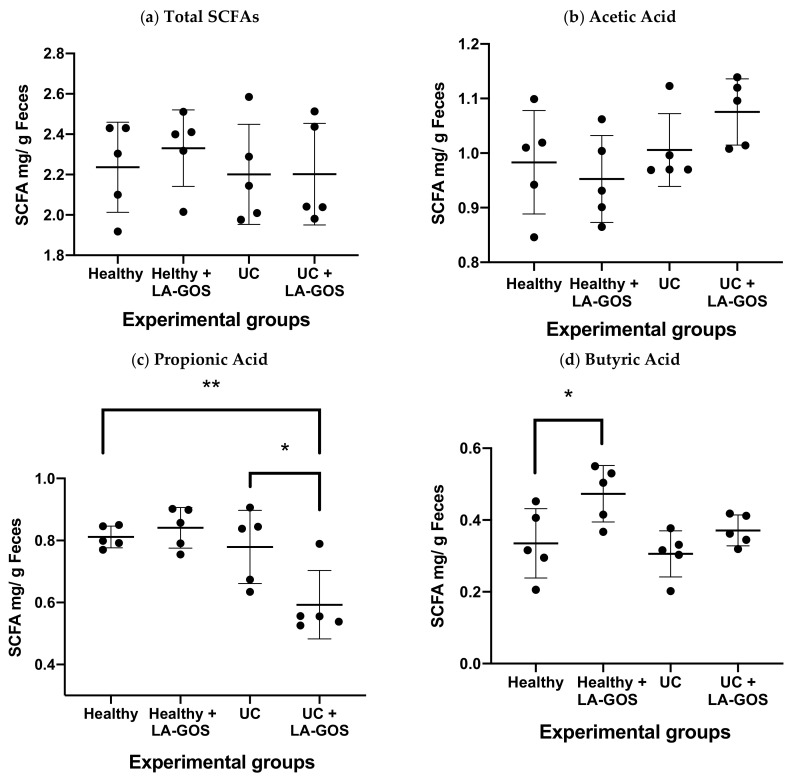
Short Chain Fatty Acid (SCFA) production was evaluated in the groups with and without LA-GOS treatment. The concentration of SCFAs was measured in cecum stool samples of each experimental group. SCFA concentration values are expressed as SCFA mg per gram of feces. (**a**) Total SCFAs, (**b**) Acetic Acid, (**c**) Propionic Acid, (**d**) Butyric Acid. LA-GOS treatment reduced the propionic acid level when compared the healthy and UC + LA-GOS. Indeed, the LA-GOS treatment reduced the propionic acid level when was compared against untreated UC group. On the other hand, the production of butyric acid increased in the healthy + LA-GOS group in comparison with the healthy untreated group. Data are expressed as the mean ± SD. Mean values were compared using one way ANOVA followed by Dunnett’s multiple comparison tests. (* *p* ≤ 0.05, ** *p* = 0.005).

SCFAs are the main metabolites produced by the gut microbiota that participate in the intestinal health regulation [7]. To investigate the impact of LA-GOS treatment on SCFAs, we decided to quantify the acetic, propionic, and butyric acid concentrations as well as the total SCFA levels. We found that the LA-GOS treatment did not modify the SCFA total levels or the acetic acid production (Figure 6a,b). However, in the UC + LA-GOS group, we observed a tendency to increase the acetic acid concentration. 

On the other hand, the UC + LA-GOS group showed significant lower propionic acid levels when it was compared with the healthy and UC groups (27% and 23% of reduction, respectively) (Figure 6c, * *p* = 0.01 and ** *p* = 0.005). Added to that, the butyric acid concentration was significantly augmented after LA-GOS administration in the treated healthy group (* p = 0.05), and a tendency to increase these levels was only observed in the UC + LA-GOS group (Figure 6d).

### 3.7. Treatment with LA-GOS Decreased Fecal Cecum pH Values

**Figure 7:**
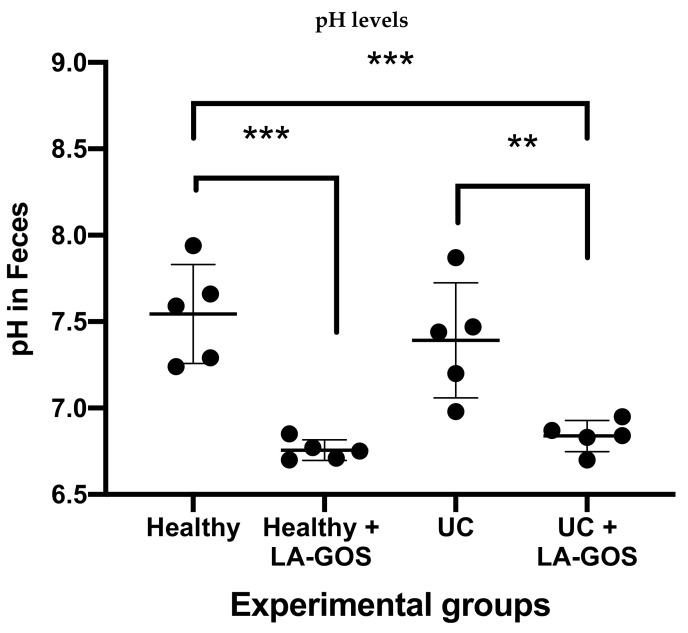
The measurement of pH was performed from cecum stool samples in each experimental group. LA-GOS treatment reduced the pH values in stool samples in comparison with healthy and UC groups. Additionally, the LA-GOS treatment caused a reduction in the pH level from UC animals in comparison with untreated UC mice. Data are expressed as the mean ± SD and statistically analyzed via one way ANOVA test followed by Dunnett’s multiple comparison test. (** *p* ≤ 0.005 and *** *p* ≤ 0.001).

Intestinal bacteria utilize the undigested fiber such GOS to generate SCFAs [69]. The SCFA production has been related with the intestinal pH levels [7]. In this study, we measured the intestinal pH from the cecum feces in all experimental groups. Our results showed a reduction on the pH levels when LA-GOS treatment was administered (Figure 7). Notably, we found a significant difference when pH values were compared between both healthy groups (7.54 ± 0.29 versus 6.76 ± 0.06, *p* ≤ 0.001). In the same manner, the UC + LA-GOS group also exhibits a lower pH in comparison with the untreated UC group (6.83 ± 0.09 versus 7.40 ± 0.33, *p* ≤ 0.005). We demonstrate that the LA-GOS treatment promoted a lower pH in feces, which is related with the SCFA production and intestinal colonic health [69].

## 4. Discussion

*Lupinus albus* is an important legume in animal and human nutrition due to the high content of protein and other compounds, including fiber and a high proportion of GOS [39,70]. Studies have demonstrated the prebiotic potential of GOS with increases of *Bifidobacterium* and *Lactobacillus* in in vivo and in vitro models [40,48,71]. In addition, the prebiotic effect is not only in the site of utilization, GOS have been studied for their prebiotics benefits in the lungs and brain [72]. 

In recent years, prebiotics have taken great relevance in the complementary treatment of several diseases, such as obesity, colon cancer, and inflammatory bowel disease [73,74,75]. However, the effect of GOS from *Lupinus albus* (LA-GOS) has not been investigated in ulcerative colitis. Since ulcerative colitis is a frequent disease in the modern lifestyle, the use of vegetal compounds like LA-GOS could be an alternative to complement the management in a natural way.

The results of this study showed that LA-GOS treatment significantly reduced certain histopathological characteristics developed by the ulcerative colitis as disease activity index, the cellular infiltration in the mucosa, and submucosa and intestinal inflammation. The intestinal epithelium possesses a mucus layer that avoids the pathogen translocation and is important to maintain the colonic epithelium health. Goblet cells are the main cellular source of mucus production. Therefore, a higher number of goblet cells is desirable to ameliorate the severity of ulcerative colitis [76]. LA-GOS administration maintains the length of colon and the integrity of the crypt, reducing the extensive ulcerations and increasing the number of goblet cells. 

These results can be compared with those founded by Dai and colleagues in 2017, where Raffinose Oligosaccharides (RFOs) from soybean were extracted, enzymatically modified, and used in a DSS-induced colitis model [77]. Interestingly, our data show a better improvement in the reduction of the histopathological grade of severity in ulcerative colitis in comparison to soybean RFOs. 

On the other hand, a pharmacological grade compound that contains oligosaccharides (GFO^®^) (Otsuka Pharmaceutical Co., Ltd., Tokyo, Japan) was tested in an in vivo ulcerative colitis [67] and a decreased DAI, maintained length of colon, and a higher number of goblet cells were observed as in our study. However, the same results were reached with a dose 10-times lower in our case. In addition, the authors did not perform a semiquantitative analysis of the number of goblet cells. These data support that LA-GOS can be a suitable treatment in comparison with another type of oligosaccharides.

Colonic tissue is structured by a double mucus layer and a simple epithelium [78]. Intestinal epithelial cells (IECs) constitute a continuous physical barrier. Adjacent IECs are connected by tight junctions (TJ) that are associated with the transport of substances and the epithelium permeability [79]. There are three main TJ protein families: claudin, occluding, and junctional adhesion molecules. Claudins are the major determinants of TJ barrier proteins. Some members of the claudin family act by plugging the paracellular pathway, while others function as paracellular channels [80]. Specifically, claudin 1 acts predominantly as a barrier claudin [81].

Claudin 1 is located in the junctional areas as well as in the lateral membranes of crypt epithelial cells [33]. Downregulation of claudin-1 contributes to increased intestinal permeability through NF-κB pathway activation [82]. In ulcerative colitis in vivo models, it has been observed that the claudin-1 protein is significantly decreased in colonic tissue [54,83]. In agreement with these previous reports, we found a reduced claudin-1 expression in the UC model.

On the other hand, we observed an improved claudin 1 expression in the UC model after LA-GOS treatment in comparison with the untreated UC group. These results are concordant with the SCFA production by the gut microbiota, particularly with the amount of butyrate and its regulatory effect on the TJ [31,84,85]. On the other hand, other TJ proteins that are disrupted in ulcerative colitis must be considered to improve our work.

In health conditions, the gut microbiota is a complex microorganism community with several beneficial properties to the host [86]. The intestinal microbiota participates in the protection against external pathogens, in the digestion and absorption of nutrients, regulates metabolism, modulates the immune system, among other functions [87]. Recently, several studies have proven that the imbalance of intestinal microbiota is associated with multiple diseases [88,89,90].

Ulcerative colitis (UC) is a disease characterized by the proportion shift of intestinal microbiota. In comparison with healthy individuals, UC patients were observed to have a reduced microbial diversity, especially in specific phylum, like Firmicutes. On the contrary, an increase of the phyla Bacteroidetes and Proteobacteria are presented in UC [74]. Many reports have demonstrated the beneficial use of natural compounds as new therapeutic candidates of UC. In fact, oligosaccharides have been evaluated in this disease due to their prebiotic role [91,92].

In our study, we found that LA-GOS treatment in comparison with the UC untreated group increased the Bacteroidetes phylum. On the other hand, the Proteobacteria phylum was reduced in both healthy + LA-GOS and UC + LA-GOS groups. In addition, the Firmicutes phylum increases in both groups treated with LA-GOS (healthy and UC groups). This augment in the Bacteroidetes phylum after treatment correlates with the results of Xu and cols. [54] and Guo and colleagues [89]. The change of the abundance in Bacteroidetes phylum could be clinically meaningful since it has been associated with IBD in comparison with healthy individuals [89].

Although a reduction of the Firmicutes phylum was shown subsequent to the treatment in those studies, in our work, this phylum was increased through LA-GOS administration. The abundance of Firmicutes phylum is associated with specific butyrate producer bacteria, such as *Faecalibacterium prausnitzii, Roseburia* sp., and *Eubacterium rectale*. LA-GOS treatment improve the butyrate producer bacteria (Figure S2).

The Proteobacteria phylum is reduced after LA-GOS treatment, similar results were found by Guo, indicating that the low abundance of this phylum could improve dysbiosis in UC [89]. In addition, the association of an inflammatory environment is highlighted with an up-regulated proportion of Proteobacteria in UC [93]. Notwithstanding the used methodology, the reduction of total bacteria in an experimental UC model is consistent with the results found in other studies Xu and cols. [54] and Guo and colleagues [89]. Remarkably, we observed that LA-GOS treatment increased the total bacteria in comparison with the UC untreated group that is commonly associated with intestinal health [94].

The production of SCFAs has been related with prebiotics and gut microbiota. Among the SCFAs produced by the intestinal microbiota, butyrate exerts multiple health benefits to the host [95]. A wide range of human butyrate-producing strains belonging mainly to the Firmicutes phylum. In fact, butyrate is produced by two pathways: butyrate kinase/phosphotransbutyrylase (*Buk*) pathway and butyryl coenzyme A (CoA): acetate CoA transferase (*But*) pathway [62]. Here, we decided to quantify the *But* gene since it is related to the main pathway used by the microbiota [96].

It has been observed that the *But* gene is significantly decreased in fecal microbiota from patients with UC [97]. The reduction of the abundance of the *But* gene after experimental UC is in agreement with this finding. However, LA-GOS administration does not increase the *But* gene abundance after the period of treatment. In another study, it was reported that oligosaccharides administration (FOS), did not modify the *But* gene abundance in spite of a longer treatment period (9 weeks) [51].

Notably, we found a significant augment in the *But* gene abundance of the healthy + LA-GOS group in comparison with the untreated healthy group. An explanation for these results could be the impaired association between the abundance of total bacteria and the *But* gene positive bacteria. The total bacteria were reduced in both UC groups and, in the healthy groups, were maintained. Thus, in a healthy environment, LA-GOS administration promotes the abundance of *But* gene positive bacteria, while in experimental UC, it is not able to preserve this abundance in the total microbial community.

The breakdown of resistant starch and undigested carbohydrates by the gut microbiota promotes the production of SCFAs [69]. The most abundant SCFAs are acetic, propionic, and butyric acids. These three acids represent around 90–95% of the SCFA total production in the human colon [7]. These acids are involved in many physiological functions as source of energy, gene expression regulators, ligands to specific receptors in enterocytes and immune cells, participation in tight junction mechanisms, modulation of lipids and carbohydrates metabolism, and pH regulation in the intestine and feces [98,99].

The total SCFA production has been evaluated in patients with IBD, these patients show reduced levels of SCFAs in intestinal mucosa and feces [100]. The main modification of SCFAs profile in patients with UC reduction of butyric acid levels. On the other hand, many reports showed that acetic and propionic acids levels were normal or reduced in UC patients [101]. In agreement, we found that the total SCFAs and the acetic acid concentrations remained without modifications in all treated and untreated groups. The total SCFA production can be compared with the results of Ferrer and cols. in 2020 [102].

These authors did not find any significant differences between control group and Crohn Disease (CD) patients. The intestinal homeostasis is associated with the amount of SCFAs. Nevertheless, the diminution of total bacteria is associated with the down levels of SCFAs [31]. We can speculate that the total SCFAs concentration maintenance particularly in the UC group could be explained by the damage of the intestine and the down regulation in the SCFAs transport to enterocytes [103].

The acetic acid is principally produced by Bacteroidetes phylum and *Bifidobacterium* genus. The acetic acid is the major SCFAs produced and is converted into acetyl-CoA. Later, acetyl-CoA can be metabolized into the tricarboxylic acid cycle to generate energy [74]. In addition, the acetate is used by specific species of the Firmicutes phylum (*Faecalibacterium prausnitzii* and *Roseburia* sp.) to produce butyric acid through the butyryl-CoA: acetate CoA transferase pathway [68]. The 50% of butyrate producers used acetate to produce butyrate [104]. This knowledge supports the increase of the Bacteroidetes phylum as well as the acetic acid level maintenance in the UC + LA-GOS group.

The propionic acid is produced by some species of Bacteroidetes phylum, Firmicutes phylum, *Bifidobacterium* generous and *salmonella* spp. [98]. The propionic acid level was significantly reduced by the LA-GOS treatment in the UC model in comparison with healthy and UC groups. In a healthy environment, propionate participates in the regulation of gluconeogenesis and cholesterol synthesis in the liver. The effect of propionate is controversial since it has been related with intestinal anti-inflammatory effects and prevention of pathogen colonization [29].

On the other hand, in patients with CD, this promotes a virulent form of the adherent-invasive *Escherichia coli* [105]. The modulation of propionate could be associated with other molecules, like the amino acids and pH level. A linear correlation has been described in relation to the propionate and pH levels [106]. Taken together, we can hypothesize that propionic acid concentration in the UC + LA-GOS treatment could be conceivably related with the low abundance of proteobacteria phylum or with the pH decrease.

Butyric acid is the most dynamic of the three mentioned SCFAs. It is the source of 70% of the energy for the intestinal epithelial cells, increases the MUC2 gene expression and mucin production, decreases cell proliferation, and enhances the epithelial barrier [107]. It is produced by the Firmicutes Phylum specifically by the following four families: *Ruminococcaceae, Lachnospiraceae, Erysipelotrichaceae,* and *Clostridiaceae* [98]. The main species related to the production of butyrate are *F. prausnitzii, Eubacterium rectale, Eubacterium Hallii,* and *Roseburia intestinali* [98,108]. In our study, the butyric acid concentration increases after LA-GOS treatment in healthy mice. Butyric acid levels could be explained by the increase in Firmicutes phylum and the *But* gene positive bacteria after LA-GOS treatment in healthy conditions.

Potentially, colonic acidity environment provides protection against colonization of pathogen bacteria (*Salmonella typhimurium, Escherichia coli, and Shigella* sp.) [109]. Moreover, pH levels are inversely associated with SCFAs concentration [30]. Accordingly, in an in vitro study, acetic acid inhibited the growth of many common pathogens, especially at a lower pH [110]. In our results, we found that LA-GOS treatment significantly reduced the colonic pH under healthy and UC conditions. In a previous study of our group, we demonstrated that the pH level is a regulator of immune cell activity, including neutrophils.

In general, neutrophils are implicated in the inflammatory process related with intestinal damage in the UC model [111]. A lower pH reduced the production of Neutrophil Extracellular Traps (NET) by neutrophils [112]. The decrease of NET could be associated with an improvement in the intestinal health and anti-inflammatory effects. These facts could support the observed health beneficial effect of pH level reduction after LA-GOS treatment (Figure S3). Our study sets the basis for future research focused on identifying bacteria families, genus and species involved in the SCFA production and the potential role of LA-GOS treatment in this intestinal ecosystem.

## 5. Conclusions

In this work, we demonstrated, for the first time, the effect of LA-GOS treatment in the reduction of DAI and inflammatory process in UC. In addition, a stratification of DAI and inflammatory process versus the number of *But* gene positive bacteria will be important to justify the use of prebiotics. Moreover, it is necessary to realize more studies to augment the number of studied animals, modulate the dosage of DSS, and include inflammatory biomarkers, such as cytokines and immune cells. The use of glucocorticoid must consider control of the inflammatory response.

## Figures and Tables

**Table 1:** Disease Activity Index (DAI).

Score	Weight Loss	Stool Consistency	Blood in Feces
0	0	Normal	Negative
1	1–5%	Soft but still formed	Thimbleful, blood steak
2	5–10%	Sof and unformed	Modicum, blood Clot [54]
3	10–20%	Lose	Visible bloody stool
4	>20%	Diarrhea	Gross bleeding
Taken from Ref. [54]			

**Table 2:** Scoring scheme for chemically induced colonic inflammation.

**Inflammatory Cell Infiltrate**		**Score 1**	**Intestinal Architecture**		**Score 2**
Severity	Extent		Epithelial change	Mucosal architecture	
Mild	Mucosa	1	Focal erosion		1
Moderate	Mucosa and Submucosa	2	Erosions	±Focal Ulcerations	2
Market	Transmural	3		Extended ulcerations ± granulation tissue ± pseudopolyps	3
Total sum of score 1 and 2 = 0 to 6					
Taken From Ref. [55]					

## Data Availability

Not applicable.

## Supplementary Materials

The following are available online at https://www.mdpi.com/article/10.3390/biom11111658/s1, Figure S1. Differents dosages of LA-GOS increase the Butyryl-CoA transferase gene positive bacteria, Figure S2. LA-GOS treatment improves the relative abundance of butyrate producer bacteria, Figure S3. Potential mechanism of LA-GOS treatment in ulcerative colitis model.
