# Supplementary material for: In Vivo Healthy Benefits of Galacto-Oligosaccharides from Lupinus albus (LA-GOS) in Butyrate Production through Intestinal Microbiota
Source: Biomolecules. 2021 Nov 9;11(11):1658. doi: 10.3390/biom11111658 (PMC8615603; doi:10.3390/biom11111658)
Supplement: Supplementary file 1 [file biomolecules-11-01658-s001.zip › biomolecules-1410463-supplementary.pdf]

Supplemental Material

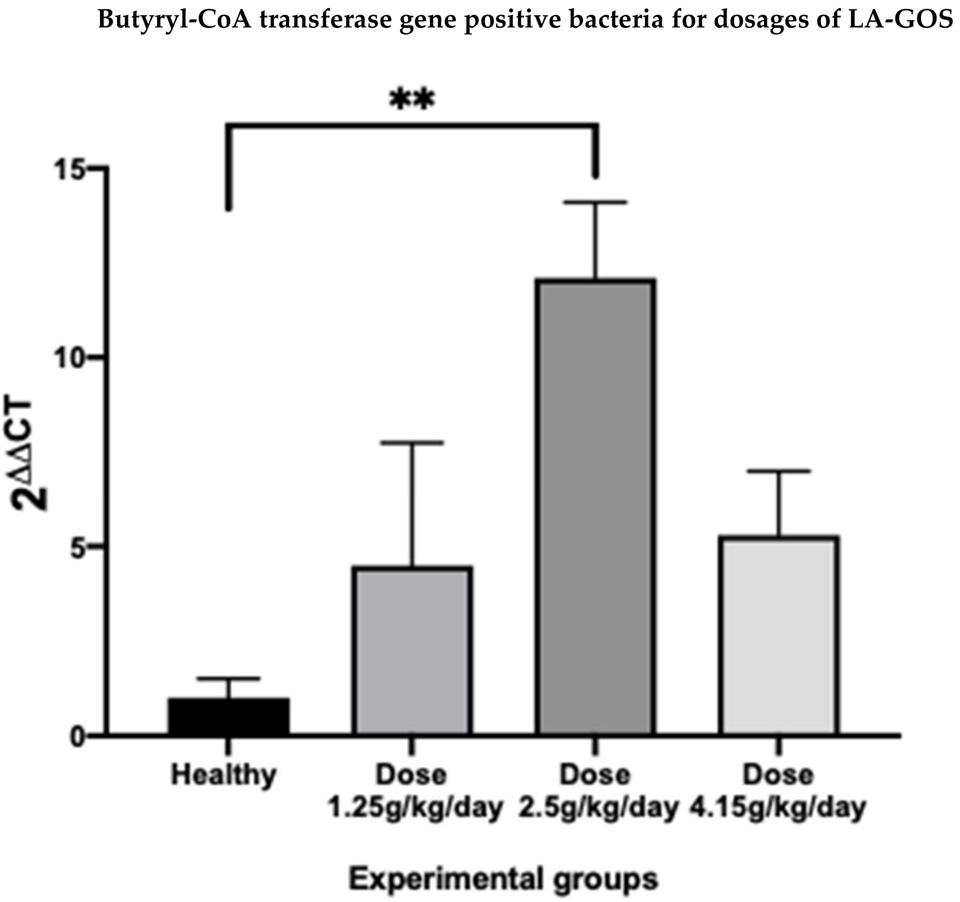

Figure S1. Different dosages of LA-GOS increase the Butyryl-CoA transferase gene positive bacteria. In healthy mice, Butyryl-CoA transferase (*But*) gene was quantified in the total bacteria in feces on the 14th day. The *But* gene was relatively quantified using $2^{\Delta\Delta CT}$ analysis. Three dosages were selected to evaluate the effect of LA-GOS treatment (1.25, 2.5, and 4.15 g/kg/d). Data analysis revealed significant differences between the healthy and the dose of 2.5 g/kg/d. Results are expressed as mean ± SEM. One way ANOVA was performed to analyze the data followed by Dunnett's multiple comparison test (** $p \leq 0.007$).

(a) ***Faecalibacterium prausnitzii*** **(b)** ***Roseburia sp.*** **(c)** ***Eubacterium rectale***

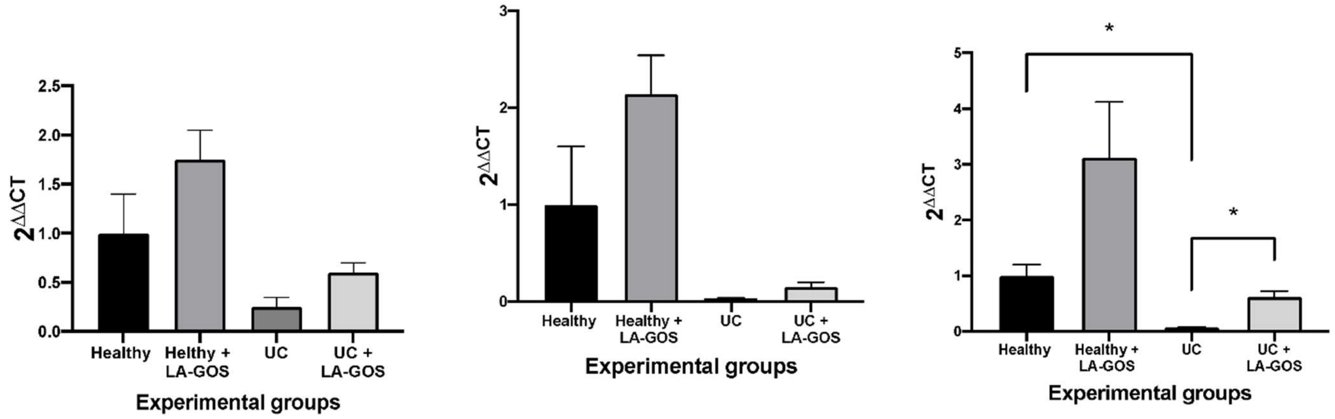

Figure S2. LA-GOS treatment improves the relative abundance of *Eubacterium rectale.* Butyrate producer bacteria *Faecalibacterium prausnitzii,Roseburia sp,* and *Eubacterium rectale* were analyzed to associate the production of butyric acid with the specific species of gut microbiota at the 14th day of treatment. The relative quantification (real-time PCR) was determined by $2^{\Delta\Delta CT}$ analysis between total bacteria and each specific butyrate producer bacteria. The sequences of each pair of primers were Fprsn_F: GACAAGGGCCGTCAGGTCTA Fprsn_R: GGACAGGCAGATRAAGCTCTTGC for *F prausnitzii*, *Roseburia sp*. and *E. rectale* share the forward sequence RosEub_F: TCAAATCMGGIGACTGGGTWGA, Ros_R: TCGATACCGGACATATGCCAKGAG for *Roseburia sp*. and Eub_R: TCATAACCGCCCATATGCCATGAG for *E. rectale.* The conditions of amplification were in accordance with the reference (Shinohara, R., 2017). (**a**) and (**b**) The administration of LA-GOS in healthy and UC groups showed a tendency to increase the proportion of *F. prausnitzii*; however, these differences were not statistically significant as in *Roseburia sp*. (**c**) The relative abundance of *E. rectale*. Statistical differences were found between healthy and UC groups, and LA-GOS treatment significantly increased the proportion of *E. rectale* in the UC treated group. The results are expressed as the mean ± SEM. One way ANOVA was performed to analyze *F. prausnitzii* and *Roseburia sp*., followed by Dunnett's multiple comparison test. The Brown–Forsythe test was performed to analyze *E. rectale*, followed by Dunnett's T3 multiple comparison test. (* $p \leq 0.05$).

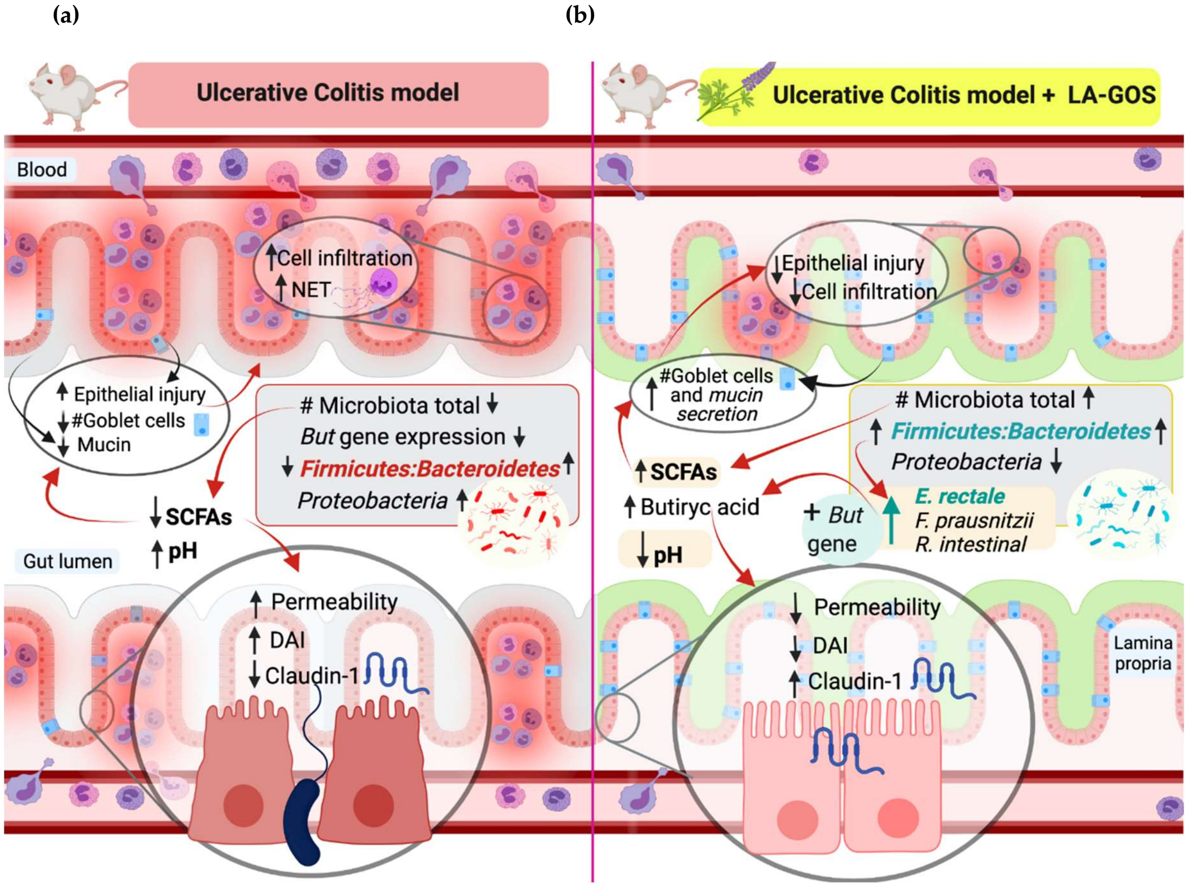

Figure S3. Potential mechanisms of LA-GOS treatment in an ulcerative colitis model. (**a**) Ulcerative colitis is a disease characterized by a decrease in diversity and an abundance of total bacteria associated with an increase in Proteobacteria and reduction of Firmicutes phylum and *Eubacterium Rectale.* These changes in microbiota reduced Short Chain Fatty Acid (SCFA) production and higher pH levels. The stability of the colonic epithelium is disrupted by the lower SCFA production, resulting in a lower number of goblet cells, production of mucin and down expression of tight junction proteins, such as claudin-1. The reduction in claudin-1 and mucin production trigger epithelium permeability, promoting the cross of gut microbiota into the inside of colonic cells, activating the immune system, including neutrophil and NET (Neutrophil Extracellular Trap) production, which produces epithelial injury and cell infiltration. (**b**) LA-GOS treatment improves the intestinal health to change the proportion of gut microbiota. The shift of microbiota by LA-GOS increases the Firmicutes phyla as well as *But* gene positive bacteria, such as *Eubacterium rectale.* SCFA production is augmented, and the pH level is acified, resulting in a healthy microenvironment to prevent pathogen colonization (Proteobacterias). The use of LA-GOS as a prebiotic causes a reduction in DAI and inflammation and is a candidate to treat this pathology.
